# Propedia: a database for protein–peptide identification based on a hybrid clustering algorithm

**DOI:** 10.1186/s12859-020-03881-z

**Published:** 2021-01-02

**Authors:** Pedro M. Martins, Lucianna H. Santos, Diego Mariano, Felippe C. Queiroz, Luana L. Bastos, Isabela de S. Gomes, Pedro H. C. Fischer, Rafael E. O. Rocha, Sabrina A. Silveira, Leonardo H. F. de Lima, Mariana T. Q. de Magalhães, Maria G. A. Oliveira, Raquel C. de Melo-Minardi

**Affiliations:** 1 Laboratory of Bioinformatics and Systems (LBS), Department of Computer Science, Universidade Federal de Minas Gerais, Av Pres. Antônio Carlos, Belo Horizonte, MG 31720-901 Brazil; 2 Macromolecule Biophysics Laboratory (LBM), Department of Biochemistry and Immunology, Universidade Federal de Minas Gerais, Av Pres. Antônio Carlos, Belo Horizonte, MG 31720-901 Brazil; 3 Department of Computer Science, Universidade Federal de Viçosa, Av Peter Henry Rolfs, Viçosa, MG Brazil; 4 Department of Biochemistry and Molecular Biology, Universidade Federal de Viçosa, Av Peter Henry Rolfs, Viçosa, MG Brazil; 5 Laboratory of Molecular Modeling and Bioinformatics, Department of Exact and Biological Sciences, Universidade Federal de São João Del-Rei, Rua Sétimo Moreira Martins, Sete Lagoas, MG Brazil

**Keywords:** Database, Webserver, Protein structure, Protein–peptide complexes, Peptides, Clustering, Protein design

## Abstract

**Background:**

Protein–peptide interactions play a fundamental role in a wide variety of biological processes, such as cell signaling, regulatory networks, immune responses, and enzyme inhibition. Peptides are characterized by low toxicity and small interface areas; therefore, they are good targets for therapeutic strategies, rational drug planning and protein inhibition. Approximately 10% of the ethical pharmaceutical market is protein/peptide-based. Furthermore, it is estimated that 40% of protein interactions are mediated by peptides. Despite the fast increase in the volume of biological data, particularly on sequences and structures, there remains a lack of broad and comprehensive protein–peptide databases and tools that allow the retrieval, characterization and understanding of protein–peptide recognition and consequently support peptide design.

**Results:**

We introduce Propedia, a comprehensive and up-to-date database with a web interface that permits clustering, searching and visualizing of protein–peptide complexes according to varied criteria. Propedia comprises over 19,000 high-resolution structures from the Protein Data Bank including structural and sequence information from protein–peptide complexes. The main advantage of Propedia over other peptide databases is that it allows a more comprehensive analysis of similarity and redundancy. It was constructed based on a hybrid clustering algorithm that compares and groups peptides by sequences, interface structures and binding sites. Propedia is available through a graphical, user-friendly and functional interface where users can retrieve, and analyze complexes and download each search data set. We performed case studies and verified that the utility of Propedia scores to rank promissing interacting peptides. In a study involving predicting peptides to inhibit SARS-CoV-2 main protease, we showed that Propedia scores related to similarity between different peptide complexes with SARS-CoV-2 main protease are in agreement with molecular dynamics free energy calculation.

**Conclusions:**

Propedia is a database and tool to support structure-based rational design of peptides for special purposes. Protein–peptide interactions can be useful to predict, classifying and scoring complexes or for designing new molecules as well. Propedia is up-to-date as a ready-to-use webserver with a friendly and resourceful interface and is available at: https://bioinfo.dcc.ufmg.br/propedia

## Background

Peptides are short chains of amino acid residues connected by peptide bonds that act in cell signaling and as immune modulators, among other important functions. It is estimated that between 15 and 40% of all protein–protein interactions in cells are mediated by these molecules [1]. Additionally, peptides are structurally diverse, versatile, induce low resistance with limited nontarget activity and can be modulated to interact with specific cellular targets, making them good therapeutic agents [2]. However, their short half-life and poor oral bioavailability has discouraged the search for peptides as therapeutics in the past [3].

With the recent emergence of new synthetic approaches that permit changes in the biophysical and biochemical properties of peptides, these molecules are once again being considered as drug candidates [4–6]. In fact, over 60 peptide drugs have been approved in major pharmaceutical markets and hundreds of others are in active clinical development at the moment [3]. Peptide-like inhibitors are used as well to treat cancer, diabetes, and autoimmune diseases and have high success rates in commercial development [7]. Multiple next-generation drug candidates (derived from exenatide, a synthetic form of a natural 39-amino acid peptide secreted by *Heloderma suspectum*), have been proposed as therapeutic agents for type 2 diabetes mellitus [3].

Understanding the structure and recognition of protein–peptide complexes may aid the design of novel peptides and peptide-based compounds for drug development or biotechnological purposes. Databases of protein–peptide complexes can pave the way for the analysis and comprehension of the mechanisms of protein–peptide recognition. There are several peptide databases, with varied purposes, as databases of bioactive peptides [8], antimicrobials [9], cell penetrating peptides [10], hemolytic peptides [11], etc. [8]. Here, we briefly review some representative examples of protein–peptide databases.

London and colleagues [12] in 2010 proposed PeptiDB, comprising 103 high-resolution peptide-protein complex structures. It was proposed as a nonredundant set of high resolution complexes to investigate the structural bases of interactions between proteins and peptides and to improve understanding binding strategies for short peptides (5–15 residues).

Also in 2010, Vanhee et al. [13] devised PepX, comprising protein–peptide complexes clustered based on binding interfaces. It was updated in 2014 for the last time (505 unique protein–peptide interface clusters from 1431 complexes) and is not available anymore.

Das et al., in turn, proposed PepBind [14] in 2013 as a curated set of 3100 protein–peptide complexes clustered according to structure determination methods and manually curated for cellular activity of complexes. The authors mentioned that there was a web interface but it seems to no longer be available.

More recently, in 2018, Frappier et al.  [15] presented PixelDB a database that comprises 1966 non-redundant high-resolution complexes. Entries are clustered based on structural similarities of receptors and then on binding modes. The authors claim to identify conserved peptide core structural motifs. We found a version of this database on GitHub updated 3 years ago.

Wen et al. [16] released PepBDB also in 2018 and this database is available through a web interface and for download. It contains 13,299 complexes and was last updated in March 2020. The web interface presents the whole list and an individual interactive visualization of the 3D interface and a 2D plot of hydrogen bonds and hydrophobic interactions using LigPlot [17]. Protein–peptide complexes can be filtered considering sequence features, structure resolution and experimental method.

At the end of 2019, Xu et al. [18] proposed PepPro, a nonredundant benchmarking tool for testing peptide-protein docking algorithms composed of only 89 complexes. For 58 complexes, the unbound protein structures are available, which is useful for evaluating to what extent docking algorithms can accommodate binding-related protein conformational changes.

In summary, a variety of databases have been proposed to explore and increase the understanding of protein–peptide interactions. Nevertheless, despite their relevant contributions when released, most of them are obsolete and/or no longer supported. Among those mentioned, PepBDB is the most comprehensive, as it contains approximately 13,000 complexes. In addition, it is the only one that provides features for binding mode analysis.

To fill these gaps, aiming at automatically collecting a broad and up-to-date data-set of protein–peptide complex structures as a useful resource for diverse peptide studies, we propose Propedia. This database is a comprehensive, general purpose and up-to-date protein–peptide resource that contains over 19,000 high-resolution structures from the Protein Data Bank (PDB) segmented in clusters to reduce redundancy if desired. Structures of complexes have been organized, facilitating search and visualization by different criteria such as PDB id, sequence similarity, peptide classification, source organism, binding area, molecular weight, aromaticity, instability index, isoelectric point, and hydrophobicity, among other computed data. These clusters not only help accommodate redundancy in the database but also allow comparisons among sequences, interfaces, interactions and functions. Therefore, Propedia is a comprehensive and powerful tool for structural studies of protein–peptide recognition, support for construction of training and test data sets for docking and scoring approaches, and facilitation of peptide rational design.

Propedia was inspired by our previous work on defense of plants against insects and pathogens. Soybean, when injured by the caterpillar *Anticarsia gemmatalis* Hübner, produces the Kunitz trypsin inhibitor (KTI) and the Bowman-Birk inhibitor (BBI), which impede protease-catalyzed degradation in the insect gut [19, 20]. Based on these inhibitors that are naturally produced by soybean, we are interested in proposing peptide or mimetic peptide molecules to inhibit the proteases of the caterpillar gut. We believe these molecules have the potential to be used in the ecological control of this pest insect. We formerly designed peptides manually, with the support of certain bioinformatic tools. Now we are investing in the development of automatic tools to support this process, such as ppiGReMLIN [21]. In this context, Propedia aims to deliver a comprehensive data set of experimental protein–peptide complexes organized in three types of clusters based on : (1) sequence similarity; (2) interface structure; and (3) protein–peptide binding site. It permits analysis of structures under different perspectives, supporting the detection of potential peptides for interacting with a target of interest, for example, peptides that are likely to inhibit proteases of the caterpillar gut. It is important to note that our database is not specific to soybean and its insect pest *Anticarsia gemmatalis* Hübner and can be applied to other data sets involving protein–peptide complexes.

## Construction and content

In this section, we detail the project decisions and the design process followed to build Propedia as well as the contents of the database.

### Database construction

We used the following criteria to retrieve PDB entries: (1) structures composed by two or more chains, (2) one chain with at least 2 and no more than 50 residues (for peptides), and (3) structures solved by NMR or X-ray crystallography with resolution below 2.5 Å. The present release is composed of 19,813 complexes (May 02, 2020). We developed in-house Python scripts and the Biopython library [22] to extract PDB data and populate the database. Each file was filtered to remove hydrogen atoms, water molecules, alternative positions [23] and crystallographic artifacts [24].

We identified protein–peptide complexes from the remaining files. Chains with lengths of 2–50 residues were classified as “peptides”. The reason for this choice is to keep Propedia comprehensive comprising the ranges used by the existing databases. Chains with more than 60 residues were classified as “receptors”. This decision was empirical since, by allowing smaller receptors, we observed complexes involving two peptides (or small unstructured proteins).

The protein–peptide interfaces were computed as follows: if there was at least one peptide atom at a distance of 6Å from any receptor atom and the protein–peptide complex had an interface area (greater than 0), then the protein–peptide complex was included in the database. We used the method of Lee and Richards [25] to compute the interface area (IA) and the accessible surface area (ASA). This algorithm returns the surface area of a protein in Å$^{2}$ and was computed by NACCESS [26] software. The software receives a PDB file as input and returns the ASA of each atom. The IA was calculated using the following equation:

1 $$\begin{aligned} IA = (ASA(A) + ASA(B)) - ASA(AB) \end{aligned}$$

where *ASA*(*A*) and *ASA*(*B*) are the ASA of the protein (A) and peptide (B), respectively, while *ASA*(*AB*) is the protein–peptide complex (AB) ASA. Then, IA is assumed to be the set of atoms that gained solvent accessibility.

With this procedure, we identified 19,813 complexes, including 19,177 from X-ray structures and 636 by NMR. There were peptides missing residues or containing nonstandard amino acid residues or binding with multiple chains describes in Table 1. Peptides bound with multiple receptors may affect both it is structural conformation and those interface residues. Therefore, we removed these complexes, obtaining 5971 protein–peptide complexes and, from now on, we refer to them as the Clusterable Complex Dataset (CCD).

**Table 1:** Summary of the number of complexes identified, by complexes with only standard amino acid residues peptides and binding with multiple receptors chains

# of receptors bound		# of complexes with only standard
with peptide	# of complexes	amino acid residues peptides
1	8.990	5.971
2	7.040	4.232
3	2.205	1.449
4	1.204	656
5	290	50
6	84	84
Total	19.813	12.442

**Fig. 1:**
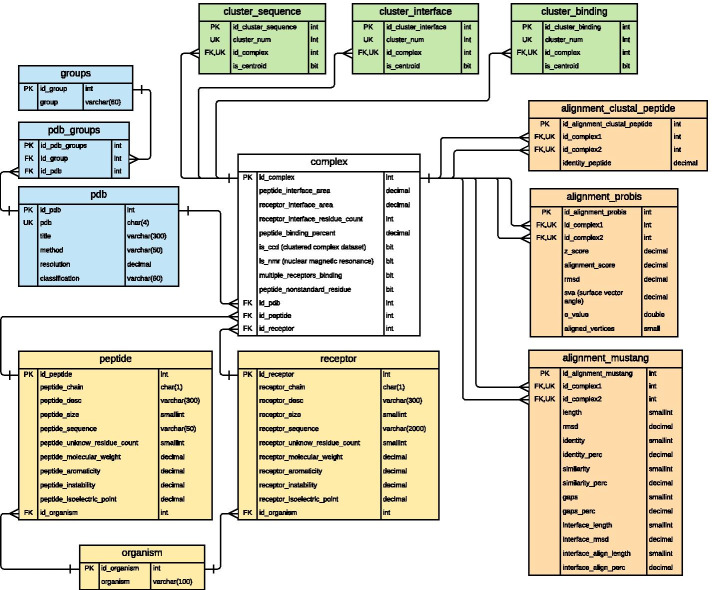
Propedia database schema, presenting the tables, fields and relationships. The complex table (white) is the core of the database and interconnects all the data; pdb entities (blue) including group, pdb_groups and pdb tables; peptide/receptor and organism tables (yellow); cluster tables (green); and alignment tables (orange)

Data collected in previous steps and computed clusters were stored in a MySQL database. The entity-relationship model is depicted in Fig. 1. We have the following entities: pdb, complex, peptide, receptor, organism, cluster (three types: sequence, interface, binding site) and alignment (clustal (peptide sequence), mustang, probis). The group table contains keywords derived from the pdb classification. For example, the Coronavirus Main Proteinase (3CLpro) (PDB id: 1p9u) is classified as ‘Hydrolase/Hydrolase Inhibitor’ and was labeled so as to be included in the groups: ‘Hydrolase’ and ‘Inhibitor’. Alignment tables store data from the results of molecular pair alignment, according to the type of clustering, and therefore have double foreign keys (id_complex1, id_complex2) corresponding with the complex table due to efficiency requirements.

**Fig. 2:**
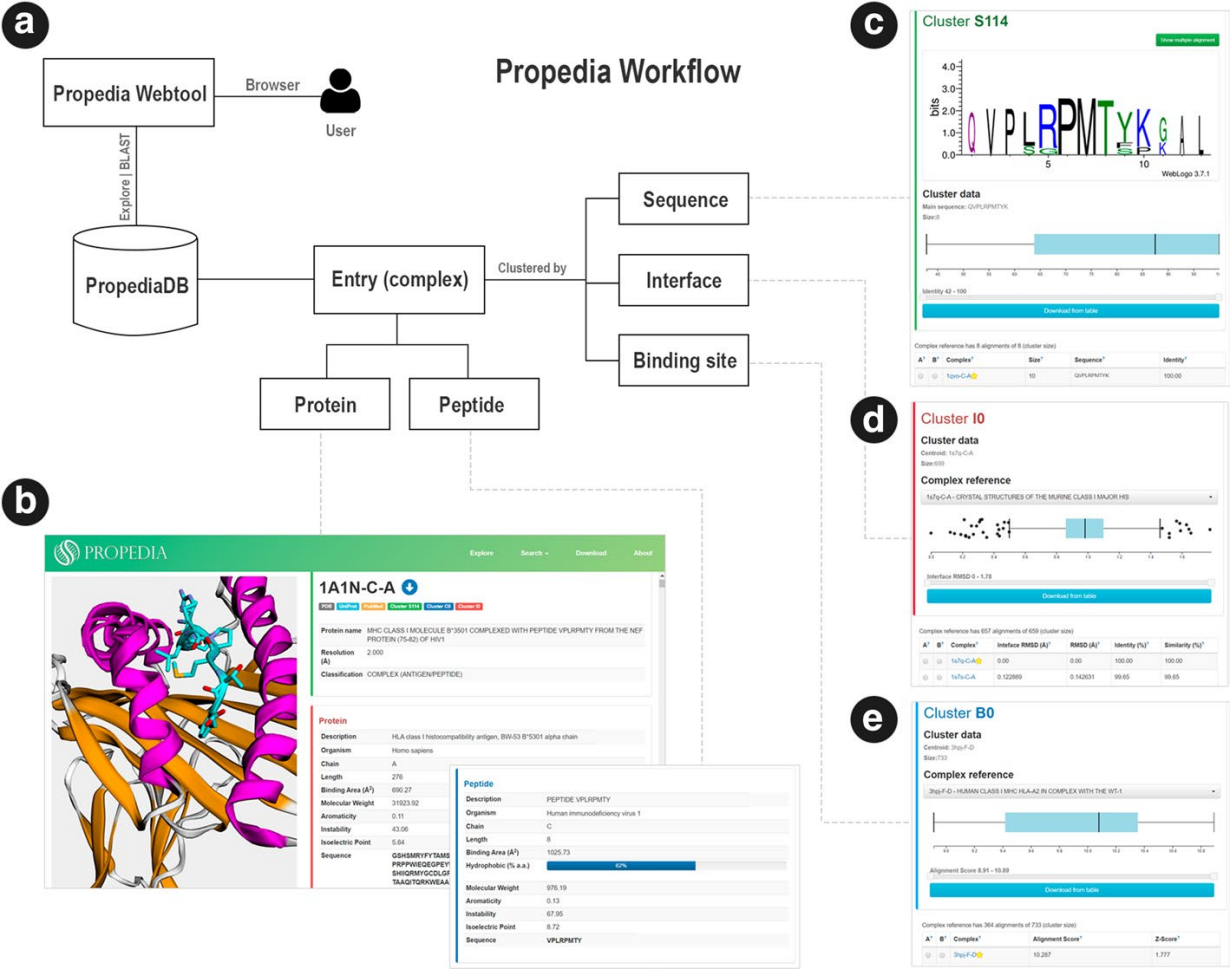
**a** Propedia scheme. The user accesses Propedia through a browser. Propedia presents each protein–peptide as a complex. Each complex can be associated with a cluster based on sequence, interface or binding site. **b** Propedia interface. Three-dimensional structure visualization of a complex. Protein is shown as a cartoon (alpha-helix in magenta and beta-strands in orange). The peptide is shown as a cartoon with cyan sticks. Complex information includes receptor features, peptide features, clustering classification and similar complexes. **c**–**e** Sequence, interface and binding site, cluster pages. Sequence cluster containing the sequence WebLogo (consensus) and main sequence. Each cluster page has a distribution chart (boxplot), used to filter complexes, according to the attributes used for clustering: sequence identity, iRMSD and alignment score

### Clustering

#### Sequences

We classified peptide sequences using the tool Hammock (1.2.0) [27]. It uses hidden Markov model profiles for peptide sequence clustering and three external tools for multiple alignments, similarity search, and HMM-HMM comparison: Clustal Omega [28, 29], HMMER 3.0 [30], and HHSuite [31]. We ran Hammock using mode ‘full’ with default parameters with the exception of ‘–min_conserved_positions’, which was set to 3, and ‘–count_threshold’, which was set to 300. These values were set empirically. Sequence labels were assigned using Python in-house scripts. CCD was used as input and after the filtering step, Hammock returned 3,495 unique sequences and classified them into 771 clusters and 1074 unique clusters (singletons), totaling 1845 peptide sequence clusters. For each cluster (non singletons) a consensus sequence was generated using the WebLogo tool [32], and the sequence alignment was determined using Clustal Omega [28, 29] to store the sequence identity among the peptides of each cluster. Centroids were identified as the peptides having the same sequence as the main sequence of each cluster.

#### Interface

Protein–peptide interfaces were aligned with MUSTANG [33], a multiple protein structural alignment tool that superposes structures using the distances of the C-$\alpha$ coordinates of residues. A pairwise structural alignment was performed using only the protein structures of the CCD. To avoid unfavorable pairwise alignments, we considered only pairs of receptors sharing over 50% sequence identity. A total of 353,545 alignments were performed in parallel in a multicore processor, and the interface RMSD (iRMSD) was calculated from the results. The protein–peptide interface was considered to be all residues within 6 Å of a peptide [34, 35]. In-house Python scripts were developed to create an undirected graph network using the NetworkX (version 1.11) Python library [36]. Nodes representing receptors and the edges (with the iRMSD between them) were added if 75% of the residues that composed the interfaces were aligned and had C$\alpha$ distance less than or equal to 2 Å. This threshold is the same for PepX [13]. Each connected subgraph from the undirected graph was considered a cluster. Altogether, 535 clusters were formed, plus 1356 singletons, for a total of 1891 non redundant protein–peptide interfaces. Each centroid was defined as the receptor node with the highest degree, and in the case of a tie, the one with the lowest sum of iRMSDs.

#### Binding sites

We used the ProBiS algorithm [37] to identify similar protein–peptide binding sites. ProBis is a local alignment algorithm that aligns similar binding sites in proteins with dissimilar folds through 3D patterns of physicochemical properties of their surfaces, considering geometrical and functional groups. Functional groups are specific groups of atoms in residues with particular physicochemical properties, which include hydrogen bond acceptors, hydrogen bond donors, acceptor/donors, aromatics and aliphatic groups [38]. ProBiS returns an alignment score for each pairwise alignment. The higher the alignment score, the more similar the binding sites are. It also computes a Z-score, a statistical measure, based on alignment scores of the population. This parameter is calculated using the Karlin-Altschul equation [39]. The input we supplied to Propedia was the CCD and we extracted surface structural patches of each receptor at a distance of 6 Å from the corresponding peptide. Then, we performed a pairwise alignment using ProBiS.

2 $$\begin{aligned} Z\_score = \frac{alignment\_score - \mu }{\sigma } \end{aligned}$$

The population mean ($\mu$) and population standard deviation ($\sigma$) were computed from pairwise alignment scores in the CCD, where $\mu$ and $\sigma$ are 1.488 and 4.951, respectively.

We used a similar method to define the clusters based on interfaces. An edge with alignment score, as weight, between two nodes (receptors) was created if the Z-score between them was greater than 1.5. This value was estimated as the point at which the number of clusters starts to increase exponentially. Connected subgraphs defined each cluster, and centroids were selected in the same way we described for previous clusters. Finally, 521 clusters and 945 singletons were generated, totaling 1466 distinct binding sites.

### Propedia webserver

The propedia database can be accessed through an interactive webserver implemented in the CodeIgniter PHP framework. Graph visualizations were implemented with the D3.js library (https://d3js.org). Protein–peptide three-dimensional structure visualizations were generated using the 3Dmol.js library [40]. The receptor/peptide sequence search mechanism is based on the blastp tool from NCBI-BLAST+ suite [41, 42] and for the binding site search we use the ProBiS algorithm [37].

## Utility and discussion

The Propedia interface (https://bioinfo.dcc.ufmg.br/propedia) is user-friendly, visual and interactive. It allows database searches with several options (Fig. 2a). Each entry in Propedia represents a protein–peptide complex. The web tool allows access to entries through PDB id, which can be followed (optionally) by protein chain id and peptide chain id.

**Fig. 3:**
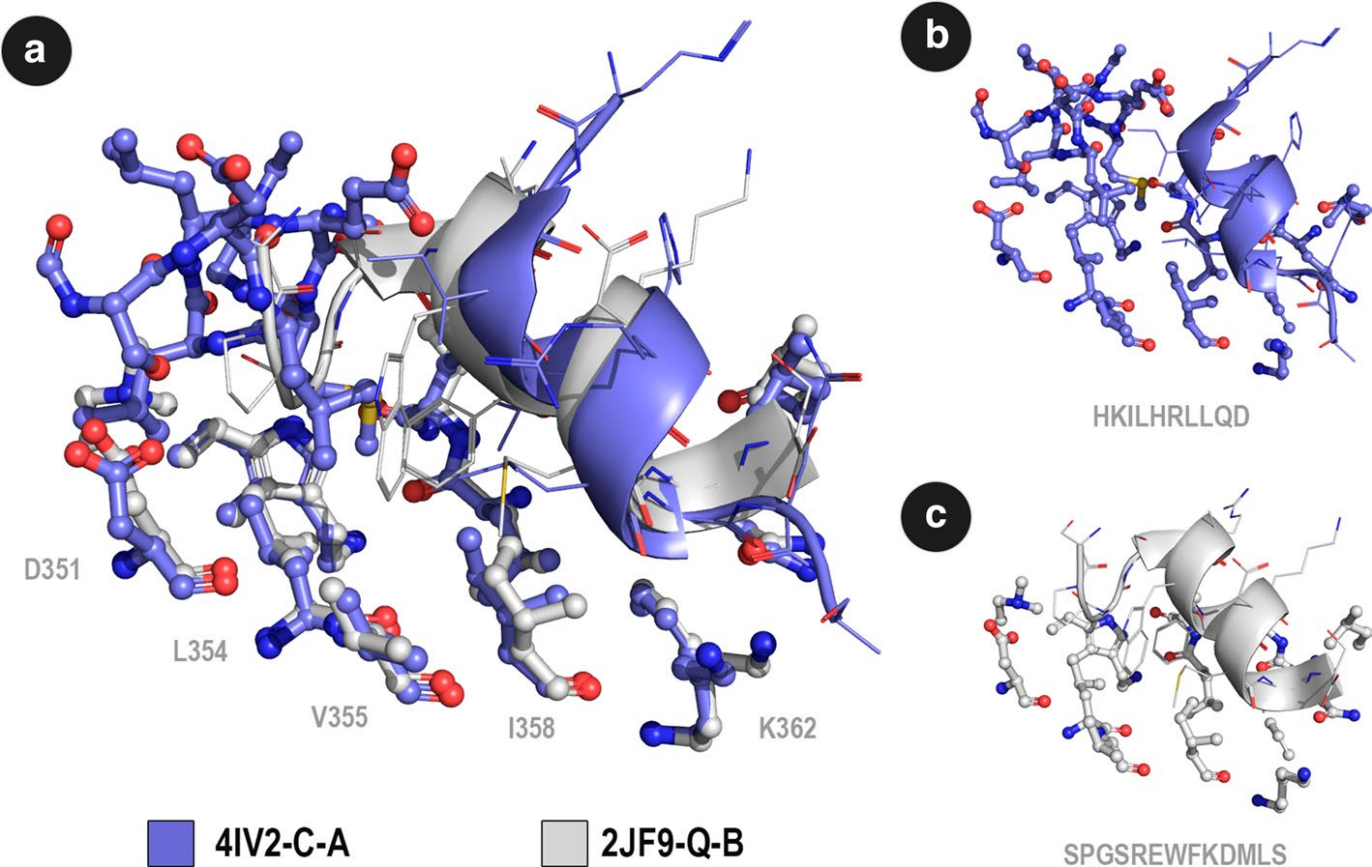
**a** Structural alignment between 2JF9 and 4IV2. The protein residues were conserved, but the peptide residues were not. **b** Estrogen receptor alpha LBD in complex with a tamoxifen-specific peptide antagonist (PDB id: 2jf9; peptide chain: Q; protein chain: B). **c** Estrogen receptor alpha ligand-binding domain in complex with dynamic way-derivative (PDB id: 4IV2; peptide chain: C; protein chain: A)

Propedia’s interface allows searching by pdb, complex id, organism, group (classification keyword), peptide and protein sizes, resolution, protein and peptide sequences (using BLAST), protein binding site (using ProBiS), and similar complexes using different clustering methods. When the user selects a particular complex to analyze, the web page presents the pdb, complex id, resolution, protein/peptide description and organism, and their data, includes chain, length, binding area (Å$^{2}$), molecular weight, hydrophobic percent (peptide only), aromaticity, instability index, isoelectric point, and sequence. We enriched Propedia with other relevant information from multiple databases such as UniProt and PubMed: protein chain, length, binding area and sequence information (Fig. 2b).

**Fig. 4:**
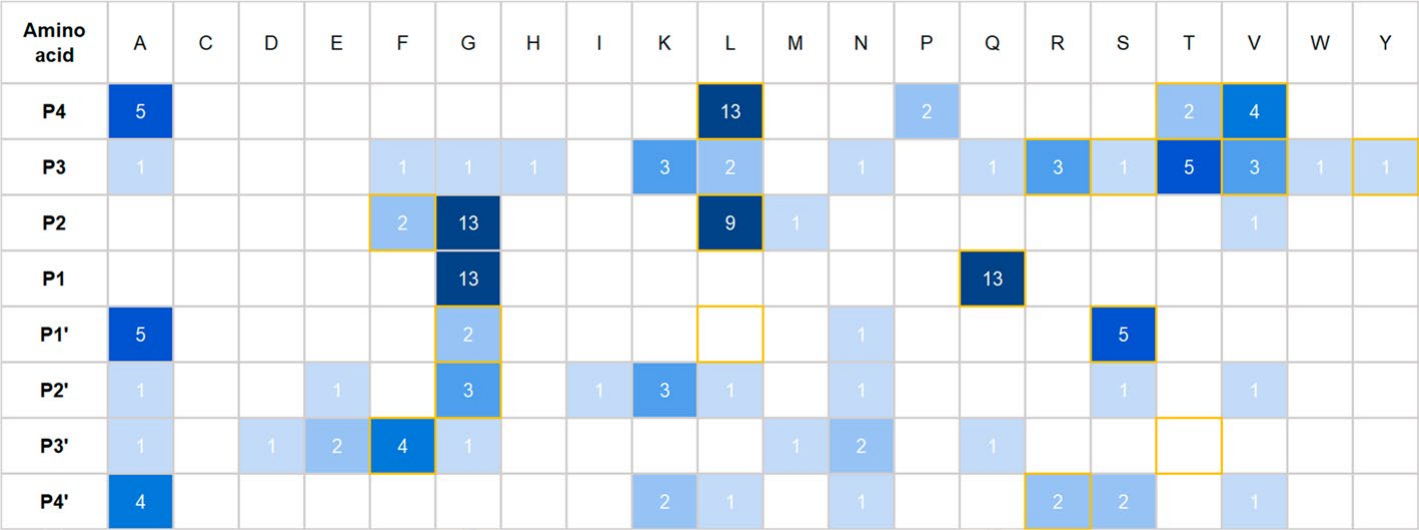
MEROPS specificity matrix in shades of blue and residues from Propedia suggested peptides highlighted in yellow

The Propedia database was built based on a hybrid clustering approach that segments the set of complexes by the following: (1) sequence similarity; (2) interface structure; and (3) protein–peptide binding site. Due to this organization in clusters, users find similar protein–peptides complexes not only by traditional sequence and/or structure conservation but by interactions as well. Interactions, in fact, are essential for molecular recognition. A user can choose among these three different approaches to eliminate redundancy of the data set, if needed.

### Comparison with other peptide databases

There are several peptide databases available. Table 2 compares some of their features. Each existing database contributes mainly to a specific piece of biological information. PepX [13] is a protein–peptide interaction database clustered by binding interfaces. It has 1,431 complexes with peptide sizes between 5 and 35 amino acids. PepBind [14] compiles structures, sequences and experimental information for protein–peptide complexes with peptides up to 35 amino acids. PeptiDB [12] comprises only 103 high-resolution complexes with peptides ranging in size from 5 to 15 amino acids. Some of these databases are not being updated and, for others, data are not even available.

PepPro [18], PixelDB [15] and PepBDB [16], on the other hand, are more recent and up-to-date efforts. They aggregate structural data from peptides up to 50 amino acids. PepPro [18] is a benchmark database built specifically for evaluation of protein–peptide docking algorithms. It contains 89 nonredundant complex structures retrieved from 1,198 high-resolution PDB entries with peptide size ranging from 5 to 30 residues. PixelDB [15] contains 1,966 nonredundant protein–peptide structures organized into clusters to provide structural conservation data for peptide binding modes. Finally, PepBDB [16] comprises 12,241 protein–peptide complex structures and their interaction information and is useful for analyzing and benchmarking docking algorithms and scoring functions.

Propedia is a more recent and fully automated database and webserver that will be updated quarterly. It is broader (comprises the entire PDB data-set) and general purpose protein–peptide analysis tool that is ready to collect, filter, clean, and compute several features and cluster data automatically always providing a comprehensive and up-to-date resource. For instance, researchers can already retrieve SARS-CoV-2 proteins along with peptides in Propedia.

**Table 2:** Comparison between propedia and other protein–peptide complex databases

Name	# of complexes	Peptide length (aa)	Resolution (Å)	Type	Availability
Propedia	19,813	2–50	< 2.5	Web server	$\checkmark$
PepX	1431	5–35	< 2.5	Web server	N.A.
PeptiDB	103	5–15	< 2.0	PDB IDs’ list	$\checkmark$
PepBind	5314	$\le$ 35	N.A	Web server	N.A.
PixelDB	1966	5–50	< 2.5	GitHub	$\checkmark$
PepBDB	12,241	< 50	N.A	Web server	$\checkmark$
PepPro	1198	5–30	< 2.5	PDB IDs’ list	$\checkmark$

*N.A* not available

### Case studies

We designed three case studies using Propedia’s varied features to exemplify possible use cases of the tool and the adjoining database.

#### Estrogen receptors in complexes with different peptides (2JF9 and 4IV2)

We performed a case study with the estrogen receptor alpha LBD in complex with a tamoxifen-specific peptide antagonist (PDB id: 2jf9; peptide: chain Q; protein: chain B). This is a *Homo sapiens* protein classified in the PDB in the transcription category. The main objective of this case study was to test if Propedia would be able to find structures with similar binding sites but with different peptide sequences.

We compared the estrogen complex with the crystal structure of the estrogen receptor alpha ligand-binding domain in complex with dynamic way-derivative (PDB id: 4IV2; peptide: chain C; protein: chain A). Although these complexes were classified in the same cluster (B1) considering their interactions, the clusters for sequence was different (Table 3).

**Table 3:** Comparison between protein and peptide characteristics of 2JF9 and 4IV2

		4IV2–C–A	2JF9–Q–B
Protein			
Chain		A	B
Length		232	210
Binding area (Å^2^)		484.85	519.30
Peptide			
Chain		C	Q
Length		10	13
Binding area (Å^2^)		559.07	547.74
Hydrophobic (% a.a.)		40%	30%
Molecular weight		1272.50	1539.71
Aromaticity		0.00	0.15
Instability index		95.31	34.72
Isoelectric point		8.76	5.79
Sequence		HKILHRLLQD	SPGSREWFKDMLS
Clusters			
Sequence cluster		S 0	S 1024
Interface cluster		I 1	I 1
Binding cluster		B 1	B 1

**Fig. 5:**
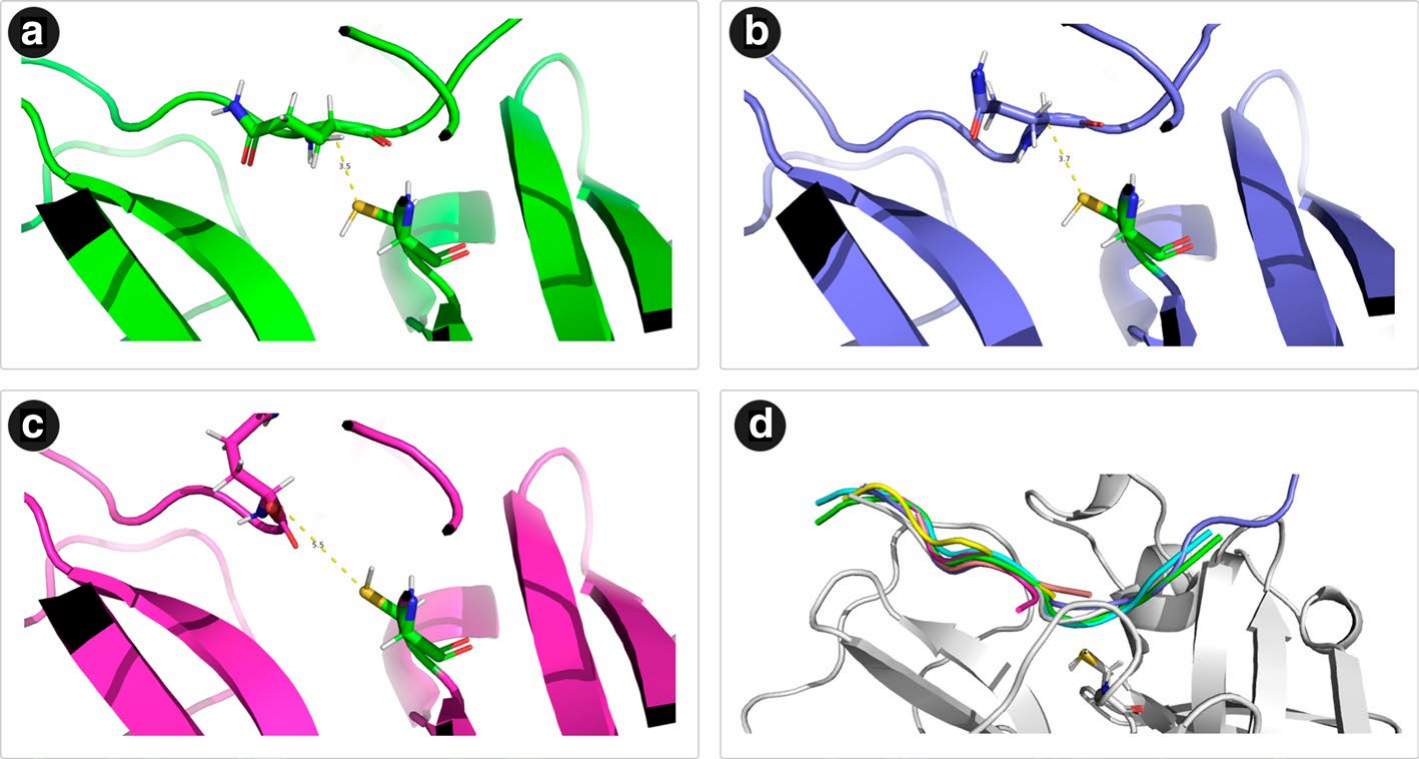
**a** PDB ID: 1lvb; peptide: chain D; protein: chain B; Rosetta score: − 538.306; Distance: 3.5 **b** PDB ID: 5om5; peptide: chain B; protein: chain A; Rosetta score: − 538.985; Distance: 3.7 **c** PDB ID: 1lvm; peptide: chain C; protein: chain A; Rosetta score: − 528.398; Distance: 5.5 **d** the whole set of evaluated peptides

Thus, we aligned the PDB files using the PyMol tool [43] and compared the results manually (Fig. 3). We observed that although the peptide primary structures were different, the peptide $\alpha$-helix folding remained the same. In addition, Propedia was able to detect similar contacts in the protein–peptide interactions, suggesting conservation in the mechanism of recognition. Additionally, our analysis showed that the protein residues were conserved, but the residues of the peptides were not. However, the interaction patterns were maintained.

This study case highlights the potential of Propedia to find similar binding patterns between proteins and peptides, even when peptide primary structure is not conserved.

#### SARS-CoV-2 main protease interactions with peptides (6LU7)

From a SARS-CoV-2 main protease structure (PDB id: 6lu7) we performed a case study to find peptides that can potentially recognize the binding site and inhibit it competitively. We submitted the 6lu7 structure to the Propedia database webserver using CCD searching scope, setting the chain (A) and binding site residues (residues within 6 Å of the N3 inhibitor). We searched for complexes with similar binding sites and the best results were ranked by alignment score (result value from the ProBiS). The top 10 results were retrieved.

From these 10 peptides retrieved in complex with similar binding sites, we obtained the peptides presented in Table 4. We verified that both of 1lvb peptides (chains C and D) have the same sequence and structural conformation. Therefore, only the complex with 1lvb-D peptide was kept for the next analyses, which has a better bound receptor (chain B), based on it is alingment score and RMSD from Propedia query results.

**Table 4:** List of retrieved peptides for SARS-CoV-2 main protease case study

PDB id	Description	Protein chain	Peptide chain	Peptide AA sequence
2q6g	SARS-CoV main protease H41A mutant	A	C	-TSAVLQSGFRK
1uk4	SARS-CoV main proteinase	B	H	—NSTLQ——-
1lvm	Thermotoga maritima methyltransferase	B	D	-ENLYFQ——-
1lvm	Thermotoga maritima methyltransferase	A	C	-ENLYFQ——-
3mmg	Tobacco vein mottling virus protease	A	C	-ETVRFQS——
1lvb	Tobacco etch virus protease	B	D	TENLYFQSGT—
1lvb	Tobacco etch virus protease	A	C	TENLYFQSGT—
5om5	Human alpha1-antichymotrypsin	A	B	-TSAVLQSGFR–
6hgj	SARS-CoV main protease variant NewBG-III	A	B	^a^
3caa	Cleaved antichymotrypsin A347R	A	B	^a^

^a^Sequences omitted due to their long length

Propedia was able to retrieve 2 proteases from SARS-CoV (previous coronavirus infecting human beings) and other viral proteases along with peptides that could be useful for the design of antiviral peptides capable of inhibiting the SARS-CoV-2 main protease. Consequently, we performed molecular docking experiments with the peptides returned by the search on SARS-CoV-2 protease. We used the Rosetta FlexPepDock docking protocol [44]. It computes high-resolution complex structures from an approximate model of a peptide within a receptor binding site, allowing full flexibility of the peptide backbone and all side chains. To provide the initial structure of each complex, we superposed the SARS-CoV-2 protease (PDB id: 6lu7) with the Propedia retrieved complex and removed the protein retaining SARS-CoV-2 and the peptide. This procedure was successful for 8 complexes, in which the SARS-CoV-2 protease was properly superposed with the model receptors (manual inspection and $RMSD \le 3$ Å). For two peptides whose receptors did not align properly due to structural high dissimilarity (PDB id: 3caa:B and 6hgj:B), we performed a global blind docking using HADDOCK [45]. Then, we selected the best model from the best cluster (most negative HADDOCK score) and submitted it as thanbe initial structure to Rosetta FlexPepDock protocol as we did with the previous 8 peptides. We had to discard both peptides (PDB id: 3caa:B and 6hgj:B) because FlexDock accommodates only peptides shorter than 30 residues. Consequently, we obtained 8 docked models, and all of them exhibited considerable affinity to the SARS-CoV-2 main protease (Table 5, column “Rosetta score”). In addition to acceptable scores, we verified apparently adequate poses (Fig. 5) of each peptide for cleavage by the site considering the proximity ($\alpha$-C of P1) to CYS145’s sulfur atom (Table 5, column “RosettaCYS distance”).

**Fig. 6:**
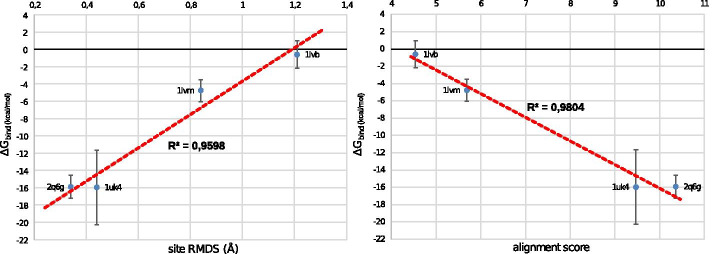
Correlation of MetaD $\Delta \hbox {G}_{{bind}}$ with site RMSD (left) and alignment score (right) from the Sars-Cov-2 $\hbox {M}_{{Pro}}$ with peptide complexes from the PDB id: 2q6g (chain C), 1uk4 (chain H), 1lvm (chain D), and 1lvb (chain D)

**Table 5:** RMSDs for SARS-CoV-2 main protease and superposition of receptors identified by propedia

PDB id	Chain	Propedia Alig. score	Propedia site RMSD	Rosetta receptors RMSD	Rosetta score	P1-CYS145 distance
2q6g	A	10.36	0.34	0.997	$-$ 542.507	3.6
1uk4	B	9.47	0.44	0.659	$-$ 525.999	3.6
1lvm	B	5.69	0.84	2.175	$-$ 525.907	4.0
1lvm	A	5.26	1.53	2.085	$-$ 528.398	5.5
3mmg	A	4.67	0.47	1.785	$-$ 530.833	3.7
1lvb	B	4.54	1.21	2.521	$-$ 530.517	3.6
5om5	A	3.18	1.63	6.665	$-$ 538.985	3.7
6hgj	A	3.38	2.20	11.156	–	–
3caa	A	3.25	2.16	9.943	–	–

In fact, according to the MEROPS database of proteolytic enzymes [46, 47], SARS coronavirus main proteases show preference for substrates of the general form: P4=V/T/A/S P3=V/W/K P2=L P1=H/Q. These positions are depicted in shades of blue in Fig. 4. According to these previous works, the site P1 is well conserved but the other sites are very mutable. The peptides identified using Propedia have residues highlighted in yellow and it can be viewed in the webserver (https://bioinfo.dcc.ufmg.br/propedia/search/binding/covid). Notice that this set of peptides is generally consistent with peptides known to inhibit SARS coronavirus proteases.

**Fig. 7:**
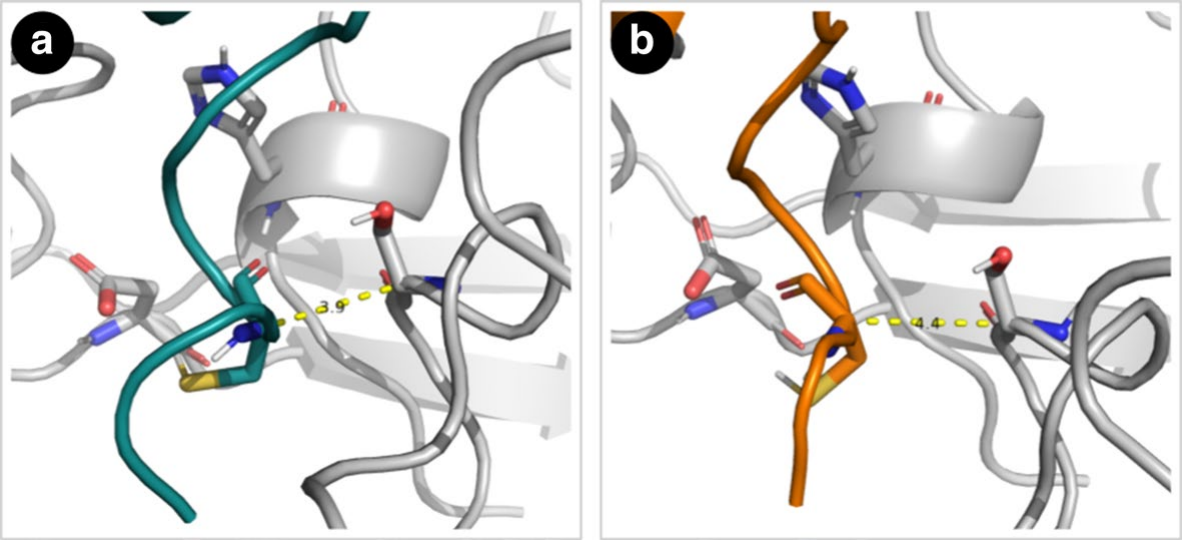
AG’s Protease model, in gray, coupled with peptides 3qgn-A (**a**) and 4dii-L (**b**). The distance between the SER143 residue from the S1 site in the protease to the cysteine residues in the peptides are 3.9 Å and 4.4 Å respectively

#### Metadynamics estimated $\Delta \hbox {G}_{{bind}}$ correlates with the major propedia scores for the Sars-Cov-2 $\hbox {M}_{{Pro}}$

The free energy landscape (FEL) for the respective triplicates of the unbinding metadynamics (MetaD) for the $\hbox {M}_{{Pro}}$: peptides complexes with the PDB id: 2q6g (chain C), 1uk4 (chain H), 1lvm (chain D), and 1lvb (chain D) are shown on Additional file 1: Figure S.1 (maps 1–3, 4–6, 7–9 and 10–12, respectively). At each system, the minima inside the protein (A) and at the aqueous environment (B) could be characterized with enough accuracy in order to estimate the binding free energy ($\Delta \hbox {G}_{{bind}}$) according the described on equations (S.1, S.2, and S.3) from the Additional file 1.

**Fig. 8:**
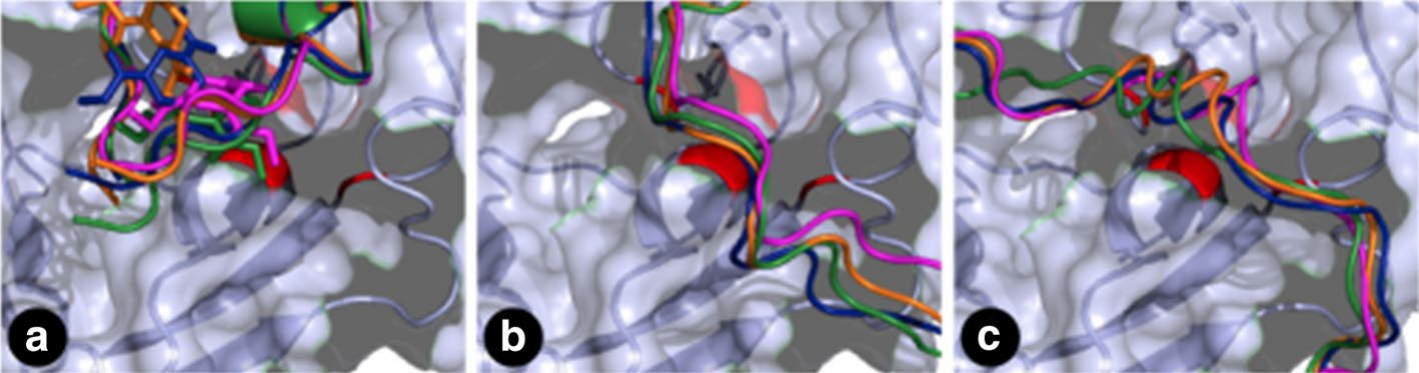
AG’s Protease model, in gray, coupled with the 4 top scored poses of peptides 6rw2-B (**a**), 3kn2-B (**b**) and 2obq-B (**c**). Residues in red represent the catalytic residues from the catalytic triad

It could be obtained a significant convergence for the MetaD recovered $\Delta \hbox {G}_{{bind}}$ values for each system in our protocol, with maximal standard deviation of 1.57 kcal$\,\hbox {mol}^{-1}$ for the systems 1lvm (chain D), 1lvb (chain D) and 2q6g (chain C) and a relatively higher deviation of 4.32 kcal$\,\hbox {mol}^{-1}$ just for 1uk4. In fact, such convergence is not surprising, once the already consolidated situation of the metadynamics technique as an accurate computational tool to estimate the binding free energy for usual ligands and peptides, being a powerful method on drug screening procedures [48–50]. The accuracy of this technique, in this way, makes it a providential instrument to validate the Propedia methodology at the screening of peptides with differential affinities for the Sars-Cov-2 MPro, given the still sparse availability of experimental data for peptide affinity at this new and important target. In this way, the correlation between the $\Delta \hbox {G}_{{bind}}$ recovered by the metadynamics higher performance method and the Propedia recovered scores was carried aiming the validation of this computational tool. It is notorious at Fig. 6 (Additional file 1: Table S.1), the significant negative correlation of the MetaD $\Delta \hbox {G}_{{bind}}$ with the Propedia recovered alignment score ($\hbox {R}^2$ of 0.98) and the positive correlation with the Propedia recovered RMSD in Å at the active site alignment procedure ($\hbox {R}^2$ of 0.96). Even, it is noteworthy that both the Propedia scores as the MetaD recovered $\Delta \hbox {G}_{{bind}}$ values put the known MPro specific substrate (PDB:2q6g) and the substrate-analogous MPro inhibitor (1uk4) at the top of the affinity ranking with this protein. In this way, both the significant correlation with the results from the high performance metadynamics method, as well the self-consistence with known functional data can be taken together as an indicative of validation for our new software, as well its applicability at the screening for functional peptides for this and other important targets.

#### Anticarsia gemmatalis protease

The velvetbean caterpillar, *Anticarsia gemmatalis* (AG) Hübner (Lepidoptera: Noctuidae) is one of the primary defoliating pests in the Americas, affecting mainly soybean crops, and a major cause of economic losses in agriculture [51, 52]. In recent years, alternative approaches towards pest control, such as the development of biopesticides, have been explored. For instance, the use of protease inhibtors is high regarded in insect pest management, as it affects the bioavailability of essential amino acids, which ultimately hinders larvae growth and the development of insects for several species, as has been shown in [53, 54].

In this case study, we used the sequence of a trypsin-like serine protease extracted from the AG’s gut, sequenced by our research group and deposited at GenBank [55] (accession JX898746.1 [56]). Additionally, a 3D model was produced using the I-TASSER server [57]. We performed a structural alignment of the model with the highest ranked templates from the modeling step in order to identify the highly conserved residues from the catalytic triad in the protease [58]. These residues were identified as HIS6, ASP56, and SER143 in the model. Additional file 1: Figure S.2 shows the superposition of the structures where the triad residues are highlighted.

We queried the Propedia database server using the protease sequence and the residues from the catalytic triad as binding site residues (along with the 3D model) in two separate experiments, and the top 10 results in each of them were selected according to their alignment scores. The results are shown in Tables 6 and 7 respectively. Then, we performed molecular docking experiments of the peptides retrieved to the model of the AG’s protease using only HADDOCK, since a considerable number of the peptides retrieved contained non-standard residues, which is not supported by PepFlexDock. Also, for the binding site query experiment, two peptides entries from the propedia results were not used (which are not listed in Table 7): 1p11-P, due to its format not being supported by HADDOCK and 3kf2-D, due to the high sequence similarity to peptide 3kf2-C in the same PDB structure.

**Table 6:** List of retrieved peptides for the AG protease case study using sequence query

PDB id	Description	Protein chain	Peptide chain
1ekb	Bovine enteropeptidase	B	A
1ekb	Bovine enteropeptidase	B	C
2stb	Salmon trypsin	I	E
2sta	Salmon trypsin	I	E
3qgn	Human thrombin	A	B
2zdv	Human thrombin	L	H
1ca8	Human thrombin	A	B
1ca8	Human thrombin	A	B
4dii	Human thrombin	L	H
4dih	Human thrombin	L	H
4lz1	Human thrombin	B	A

**Table 7:** List of retrieved peptides for the AG protease case study using binding site query

PDB id	Description	Protein chain	Peptide chain
3qgj	Lysobacter enzymogenes protease	D	C
1p11	Lysobacter enzymogenes protease	I	E
2obq	Hepacivirus NS3-4A protease	B	C
2oin	Hepacivirus NS3-4A protease R155K	C	A
2o8m	Hepacivirus NS3-4A protease S139A	D	B
3kn2	Hepacivirus NS3-4A protease	B	C
3kf2	Hepacivirus NS3-4A protease	C	A
3sga	Streptomyces griseus protease	P	E
6rw2	Human Ephrin type-A receptor 2	B	A
4a1t	Hepacivirus NS3-4A protease	D	B

For the sequence based dataset, we set the residues from the catalytic triad as active residues for the docking procedure, as well as the complete chain of each peptide. We selected the best resulting structures primarily according to the HADDOCK score (most negative) and then, according to the RMSD ($\le 3$ angstroms) of each structure relative to the overall lowest energy model. Table 8 summarize the results. Finally, peptide poses in the protease were analysed for the top 5 scored models according to the HADDOCK score, for which we identified the closest residues to the SER143 residue at the S1 site, considering the distance between C-$\alpha$ atoms. The closest residues found were cysteine residues located in models 3qgn-A (3.9 Å) , 4dii-L (4.4 Å) and 1ca8-A (5.1 Å). The presence of cysteine residues close to the serine in the catalytic tryad indicate a potential use of the peptide as an inhibitor since substrates with these residues at position P1 are not usually cleaved by trypsin-like serine proteases [59]. Figure 7 shows models 3qgn-A and 4dii-L, where the distance between residues is highlighted.

**Table 8:** HADDOCK score and RMSD for the selected models for each peptide chain in the sequence based experiment

PDB id	Chain	HADDOCK iRMSD	HADDOCK score	S1 closest residue
1ekb	C	2.564	$-$ 49.202	–
1ekb	A	2.499	$-$ 63.477	–
2stb	I	0.000	$-$ 86.803	–
2sta	I	4.275	$-$ 81.387	–
3qgn	A	0.000	$-$ 97.979	CYS (3.9 Å)
2zdv	L	0.000	$-$ 100.560	GLU (7.4 Å)
1ca8	A	1.530	$-$ 102.975	CYS (5.1 Å)
1ca8	C	1.158	$-$ 75.138	–
4dii	L	2.598	$-$ 95.836	CYS (4.4 Å)
4dih	L	1.508	$-$ 95.317	ARG (5.4 Å)

Similar to the sequence based dataset, we performed the docking for the binding site dataset using the residues from the catalytic triad, as well as complete peptide chains as active residues. A binding site signature is how a protein interacts with its ligand, and which amino acids are essential to keep the complex stable. A proper metric to verify the similarity of binding sites is the fraction of common contacts (FCC). The $FCC_{AB}$ is the ratio of contacts between structures *A* and *B* to all contacts in *A*, whose value ranges from zero, when the chains share no contacts, to a maximum of one, when all contacts of chain A are with chain B [60]. Therefore, for the binding site docking experiments, a higher average value of FCC in a cluster indicates higher similarity of the interactions between different peptide poses and the protease model, which also means that the binding site is more conserved.

For each peptide, we selected the cluster with the highest FCC score (relative to its lowest energy models produced by HADDOCK), from which sets we chose the best models according to their HADDOCK scores. FCC values and HADDOCK scores are shown in Table 9 for all peptides. The best 4 models for each of the top 3 scored clusters are shown in Fig. 8. In all models, contacts are centered in the catalytic triad (highlighted in red), while the remaining contact areas bind to different ligands, where neighboring residues on the protease side have great relevance by establishing hydrophobic and hydrogen bonds. The complete interaction map of each model is available in Additional file 1: Figure S.3. This emphasizes the importance of using FCC as a suitable metric for binding site analysis rather than RMSD, and also demonstrates Propedia’s accuracy in determining binding site patterns in regard to the ligand specificity.

**Table 9:** HADDOCK score and FCC for the selected models for each peptide chain in the binding site experiment

PDB id	Chain	Cluster FCC	Lowest HADDOCK score
3qgj	D	0.409	$-$ 34.823
1p11	I	0.621	$-$ 49.383
2obq	B	0.833	$-$ 80.258
2oin	C	0.696	$-$ 85.060
2o8m	D	0.196	$-$ 54.616
3kn2	B	0.840	$-$ 78.998
3kf2	A	0.236	$-$ 55.432
3sga	P	0.650	$-$ 61.368
6rw2	A	0.883	$-$ 75.354
4a1t	D	0.648	$-$ 87.944

## Conclusions

As far as we know, Propedia is the broadest and most comprehensive set of protein–peptide complexes. At the moment of publication of this paper, it comprises approximately 20,000 complexes. Furthermore, we developed hybrid clustering strategies that organized data into 1845 clusters based on sequences, 1891 clusters based on interface structures similarity and 1466 clusters based on binding sites. These groups may be used for detecting either nonredundant or similar complexes with several purposes going from peptide docking and scoring function benchmarking, design of biotechnological peptides and even peptide-based rational drug design. Finally, Propedia is available through a web interface, searches and analysis can be performed by a user-friendly interface and all the data are available to download.

## Supplementary information

**Additional file 1.** Additional details and figures for casestudies: Metadynamics estimated $\Delta\hbox{G}_{{bind}}$ correlateswith the major propedia scores for the Sars-Cov-2 $\hbox{M}_{{Pro}}$ and Anticarsia gemmatalis protease.

## Data Availability

The data generated and/or analyzed for current study are available at Propedia’s download page: https://bioinfo.dcc.ufmg.br/propedia/download

## Abbreviations

ASA: Accessible surface area
AG: Anticarsia gemmatalis
CCD: Clusterable Complex Dataset
FCC: Fraction of common contacts
FEL: Free energy landscape
IA: Interface area
MetaD: Metadynamics
NMR: Nuclear magnetic resonance
PDB: Protein Data Bank
RMSD: Root mean square deviation
iRMSD: Interface root mean square deviation
