# Supplementary material for: Propedia: a database for protein–peptide identification based on a hybrid clustering algorithm
Source: BMC Bioinformatics. 2021 Jan 2;22:1. doi: 10.1186/s12859-020-03881-z (PMC7776311; doi:10.1186/s12859-020-03881-z)
Supplement: Supplementary file 1 — Additional file 1. Additional details and figures for casestudies: Metadynamics estimated $\Delta\hbox{G}_{{bind}}$ΔGbind correlateswith the major propedia scores for the Sars-Cov-2 $\hbox{M}_{{Pro}}$MPro and Anticarsia gemmatalis protease. [file 12859_2020_3881_MOESM1_ESM.pdf]

ADDITIONAL FILE 1

Propedia: a database for protein-peptide identification based on a hybrid clustering algorithm

Pedro M. Martins[1], Lucianna H. Santos[1], Diego Mariano[1], Felippe C. Queiroz[3], Luana L. Bastos[1], Isabela de S. Gomes[3], Pedro H. C. Fischer[5], Rafael E. O. Rocha[1], Sabrina A. Silveira[3], Leonardo H.F. de Lima[5], Mariana T. Q. de Magalhães[2], Maria G. A. Oliveira[4] and Raquel C. de Melo-Minardi[1*]

*Correspondence: pmartinds@dcc.ufmg.br; raquelcm@dcc.ufmg.br;
[1]Laboratory of Bioinformatics and Systems (LBS), Department of Computer Science, Universidade Federal de Minas Gerais, Av Pres. Antônio Carlos, 31720-901 Belo Horizonte, MG, Brazil
Full list of author information is available at the end of the article
†Equal contributor

In this document we present additional details and figures about Propedia case studies. The document is organized in sections that are correspondent to details of metadynamics and simulations performed.

Peptide Selection for Metadynamics Validation and System Setup

Four representative peptide-protein complexes were selected from the Propedia output for the crystallographic structure of the Sars-Cov-2 main protease (the M_{Pro} with PDB:ID 6lu7) to posterior binding free energy (ΔG_{bind}) calculation by metadynamics simulation [1, 2, 3], aiming the validation of the Propedia scores. The peptides were selected looking for a maximal coverage of the Propedia ranking based both on the alignment score as at the RMSD considering the active site (Table S.1).

For the four selected complexes, the Rosetta pose presenting the best score and preserving the previously attributed protonation states was carried to a procedure of topology assembly, solvation on a water box with a 12 Å padding, and Na^+/Cl^- addition until the system neutralizing and ionic strength of 0.150 M. For these procedures, the respective psfgen, solvate and ionize tools from the VMD/NAMD packages were used [4, 5]. The CHARMM36 force field [6, 7] was used both for the protein, peptide, water and ions, as well the TIP3P model for the water molecules [8].

Simulation Procedures

All the simulations were carried at the NAMD 2.13 package [5], at the NPT ensemble, using Langevin thermostat and barostat devices, respectively set to 300 K and 1 atm. Periodic boundary conditions were used, as well particle mesh Ewald (PME) for the calculations of the long range electrostatic forces, with a 12 Å cutoff for the nonbond interactions and a 2 fs time-step. The hydrogen atoms dynamics was constrained and estimated by the SETTLE algorithm according implemented in NAMD 2.13 [5].

Before the metadynamics procedures themselves, a meticulous minimization/relaxation/equilibratio protocol was carried for each system. First, each system was minimized for 10,000 steps by conjugate gradient algorithm according implemented in NAMD 2.13 [5].

In sequence, a 10 steps relaxation/equilibration molecular dynamics (MD) protocol, at the previously listed simulation conditions and with gradual adaptation of harmonic restraints, was carried as below:

- 500 ps MD with harmonic restrains for all the atoms of the receptor and the ligand.
- 500 ps MD with harmonic restrains just for the backbone atoms of the receptor and the ligand.
- 500 ps MD with harmonic restraints just for the backbone atoms of the receptor.
- 500 ps MD without harmonic restraints.
- 8 ns MD without harmonic restraints and with previous reboot of the velocities according a 300 K and 1 atm NPT ensemble.
- 500 ps MD reintroducing the harmonic restraints at the backbone atoms of the receptor and the ligand.
- 500 ps MD reintroducing the harmonic restraints at all atoms of the receptor and the ligand.
- 300 ps MD with removal of the harmonic restraints from the ligand side chains.
- 300 ps MD with removal of the harmonic restraints from the ligand all atoms.
- 1 ns MD at the aforementioned conditions and rebooting the velocities to a 300 K and 1 atm compatible NPT ensemble.

The last 5 steps were carried in order to prepare the system for the restraints conditions used along the metadynamics procedure (i.e., harmonic restraints for the entire receptor and complete freedom just for the ligand (see below)).

After relaxation, the last frame for each system was taken to three respective and independent 10 ns metadynamics procedures (i.e., three metadynamics simulations per system) of ligand unbind from the active site according a similar protocol to the described in [3]. Basically, all the atoms of the M_{Pro} were maintained harmonically restrained, while complete dynamics freedom was given to the peptide, water molecules and ions. Two collective variables (CVs) were used to describe the unbinding process of the peptide from the catalytic pocket. The first, CV_{dist} was defined as the distance in Å between the respective centers of mass of the catalytic C145 in M_{Pro} and of the closer residue (at the starting pose) of the peptide. The respective residues for each one of the four analyzed peptides were: S7 for the PDB:2q6g peptide; Q5 for the PDB:1uk4 peptide; Q307 both for PDB:1lvm as PDB:1lvb peptides (see Results and Discussions). The second CV, CV_{ang}, was defined as the angle in degrees (º) determined by the center of mass of the M_{Pro} C145, the residue afore mentioned for each peptide and the center of mass of the peptide as a whole. The height of the Gaussians for the metadynamics was set to 0.02 Kcal/mol and added every 2ps with a width of 1.77. The CV_{dist}. ranged between 0 and 30 Å with an amplitude fluctuation of 2 Å, while the CV_{ang} has varied between 0º and 180º with an amplitude fluctuation of 10º. The potential of mean force (PMF) landscapes were saved every 1 ps. Results were analyzed with VMD software [4], in-house R and Python scripts, as well the Wordom package [9].

Free Energy Maps, Projections of the Metadynamics Energies Along the CV$_{dist}$ dimension and Estimation of ΔG$_{bind}$

To select the number of frames to be considered along the PMF reconstruction, we have used both the molecular mechanics nonbond interaction energies between the peptide and the protein, as the distance associated to CV$_{dist}$. (i.e., the distance between the respective C145 and the closer residue mass centers) as a metric to observe: 1) the access and the filling of the energy minimum A (i.e., the energy minimum for the peptide inside the protein); 2) the access and the filling of the energy minimum B (i.e., the energy minimum for the peptide outside the protein, at the aqueous environment); 3) the re-crossing event (i.e., the simulation phase in which, once the system has reached and completely filled both minima, the peptide gain higher dynamics freedom and turn to visit both minima repeatedly). Following the suggested in literature, we have selected the PMF maps saved until the simulation step immediately before the re-crossing event to reconstruct the free energy landscape (FEL) along the unbinding event [3, 10]. This is made in order to avoid the over-filling of the energy minima by the metadynamics Gaussian potentials and a loss of accuracy along such FEL reconstruction.

The CV most directly related to the unbinding process is, naturally, the one that describes the distance variation between the peptide and the active site (CV$_{dist}$). In this way, the projections of the metadynamics free energy onto the CV$_{dist}$. was calculated similarly to [3] as following equation:

$$-\beta G_{CV_{ang}}(CV_{dist}) = ln\frac{\int e^{-\beta G(CV_{dist},\ CV_{ang})}dCV_{ang}}{\int e^{-\beta G(CV_{dist},\ CV_{ang})}dCV_{ang}dCV_{dist}} \quad \text{(S.1)}$$

where $\beta = 1/\text{kbT}$, being k$_b$ the Boltzmann constant (1,9858 x 10^{-3}·kcal·mol^{-1}·K^{-1}); T = 300 K and G(CV$_{dist}$, CV$_{ang}$) accounts for the free energy value at the position (CV$_{dist}$, CV$_{ang}$) position on the PMF map.

For the estimation of ΔG$_{bind}$ from each metadynamics replica, the minima value of G$_{CV_{ang}}$(CV$_{dist}$) at a CV$_{dist}$ compatible with the complete independence of the peptide environment from the protein influence (i.e., CV$_{dist}$ $\geq$ 25 Å) was diminished from the minima value of this measure inside a distance compatible with the peptide bound (specifically or not) to the protein active site (i.e., CV$_{dist}$ $\leq$ 20 Å) according equation:

$$\Delta G_{bind} = G'_{CV_{ang}}(CV_{dist}) - G''_{CV_{ang}}(CV_{dist}) \quad \text{(S.2)}$$

where $G'_{CV_{ang}}(CV_{dist})$ is the minimum value inside, while $G''_{CV_{ang}}(CV_{dist})$ is the minimum outside the protein. For the cases in which two or more minima with similar favorability were found inside the protein, both minima were equally weighted at a global value of $G'_{CV_{ang}}(CV_{dist})$ (i.e.,$G_{CV_{ang}}(CV_{dist})^{Min}{}_{inside}$) according equation:

$$-\beta G_{CV_{Ang.}}(CV_{Dist.})^{Min.}_{inside} = ln\frac{\sum_{i=1}^{n} e^{-\beta G'_{CV_{Ang.}}(CV_{Dist.})}}{\int e^{-\beta G_{Ang.}(\ CV_{dist})}dCV_{Dist.}} \quad \text{(S.3)}$$

where i = 1, 2,..n is the number of equivalent minima at diferent CV_{dist}. values inside the protein. Finally, the accuracy of Propedia was probed by the metadynamics analysis by measuring the correlation of the ΔG_{bind} values estimated according equations (S.1, S.2, S.3) and the respective values of the alignment score and the site RMSD recovered by our tool for each one of the four M_{Pro}:peptide complexes choosen for validation.

Author details

[1]Laboratory of Bioinformatics and Systems (LBS), Department of Computer Science, Universidade Federal de Minas Gerais, Av Pres. Antônio Carlos, 31720-901 Belo Horizonte, MG, Brazil. [2]Macromolecule Biophysics Laboratory (LBM), Department of Biochemistry and Immunology, Universidade Federal de Minas Gerais, Av Pres. Antônio Carlos, 31720-901 Belo Horizonte, MG, Brazil. [3]Department of Computer Science, Universidade Federal de Viçosa, Av Peter Henry Rolfs, Viçosa, MG, Brazil. [4]Department of Biochemistry and Molecular Biology, Universidade Federal de Viçosa, Av Peter Henry Rolfs, Viçosa, MG, Brazil. [5]Laboratory of Molecular Modeling and Bioinformatics, Department of Exact and Biological Sciences, Universidade Federal de São João Del-Rei, Rua Sétimo Moreira Martins, Sete Lagoas, MG, Brazil.

References

1. Barducci, A., Bonomi, M., Parrinello, M.: Metadynamics. Wiley Interdisciplinary Reviews: Computational Molecular Science **1**(5), 826–843 (2011)
2. Bussi, G., Laio, A., Parrinello, M.: Equilibrium free energies from nonequilibrium metadynamics. Physical review letters **96**(9), 090601 (2006)
3. Brandt, A.M., Batista, P.R., Souza-Silva, F., Alves, C.R., Caffarena, E.R.: Exploring the unbinding of l eishmania (l.) amazonensis cpb derived-epitopes from h 2 mhc class i proteins. Proteins: Structure, Function, and Bioinformatics **84**(4), 473–487 (2016)
4. Humphrey, W., Dalke, A., Schulten, K., *et al.*: Vmd: visual molecular dynamics. Journal of molecular graphics **14**(1), 33–38 (1996)
5. Phillips, J.C., Braun, R., Wang, W., Gumbart, J., Tajkhorshid, E., Villa, E., Chipot, C., Skeel, R.D., Kale, L., Schulten, K.: Scalable molecular dynamics with namd. Journal of computational chemistry **26**(16), 1781–1802 (2005)
6. Huang, J., MacKerell Jr, A.D.: Charmm36 all-atom additive protein force field: Validation based on comparison to nmr data. Journal of computational chemistry **34**(25), 2135–2145 (2013)
7. Best, R.B., Zhu, X., Shim, J., Lopes, P.E., Mittal, J., Feig, M., MacKerell Jr, A.D.: Optimization of the additive charmm all-atom protein force field targeting improved sampling of the backbone ϕ, ψ and side-chain χ1 and χ2 dihedral angles. Journal of chemical theory and computation **8**(9), 3257–3273 (2012)
8. Price, D.J., Brooks III, C.L.: A modified tip3p water potential for simulation with ewald summation. The Journal of chemical physics **121**(20), 10096–10103 (2004)
9. Seeber, M., Cecchini, M., Rao, F., Settanni, G., Caflisch, A.: Wordom: a program for efficient analysis of molecular dynamics simulations. Bioinformatics **23**(19), 2625–2627 (2007)
10. Raiteri, P., Laio, A., Gervasio, F.L., Micheletti, C., Parrinello, M.: Efficient reconstruction of complex free energy landscapes by multiple walkers metadynamics. The journal of physical chemistry B **110**(8), 3533–3539 (2006)

Tables

	Propedia		MetaD		R^2 ΔG_{bind}	
PDB id	Align. score	site RMSD (Å)	ΔG_{bind} (kcal/mol)	σ	Align. score	site RMSD
2q6g	10.36	0.34	-15.92	±1.34	(-) 0.98	(+) 0.96
1uk4	9.47	0.44	-15.97	±4.32		
1lvm	5.69	0.84	-4.76	±1.27		
1lvb	4.54	1.21	-0.61	±1.57		

Table S.1 Correlation between the metadynamics estimated binding free energy (MetaD ΔG_{bind} and it is standard deviations(σ)) and the Propedia recovered alignment score (Align. Score) and site RMSD. At the last two columns the respective negative and positive correlation coefficients of the ΔG_{bind} with each Propedia parameter are depicted.

Figures

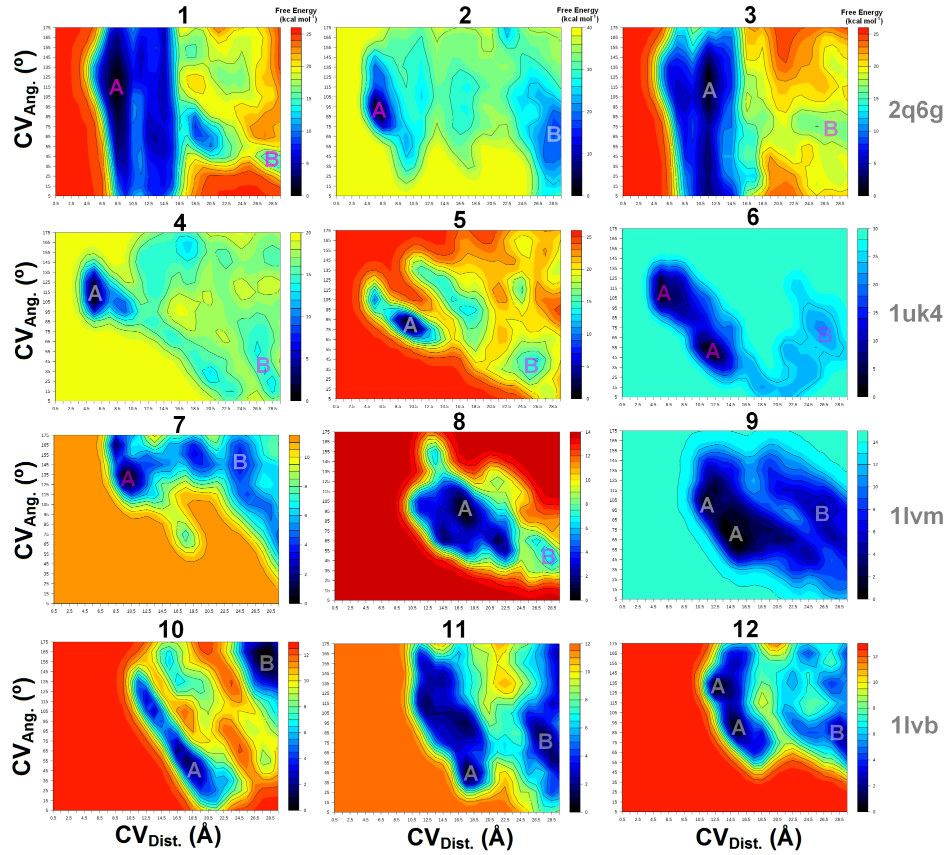

Figure S.1 The free energy landscape for the respective triplicates of the unbinding metadynamics for the Sars-Cov-2 M_{Pro}: peptide complexes with the PDB id: 2q6g(chain C), 1uk4 (chain H), 1lvm (chain D), and 1lvb (chain D)

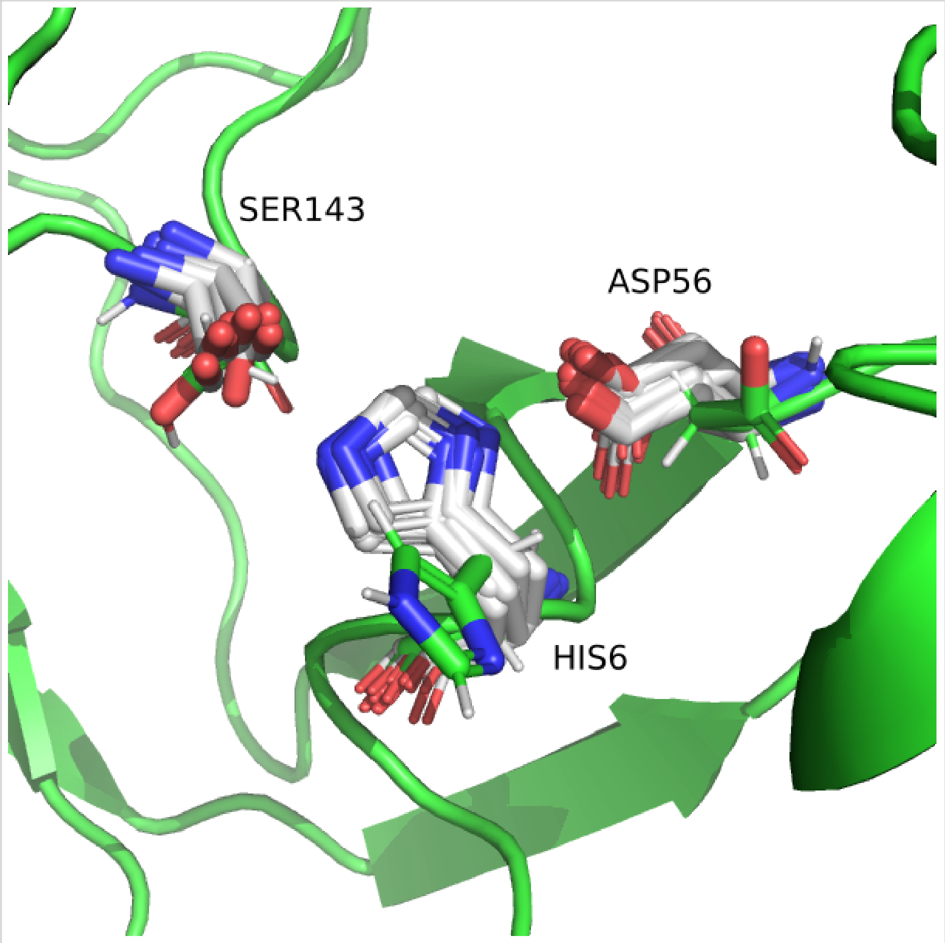

Figure S.2 Structural alignment of AG's protease model (in green), and the highest ranked templates used in the modeling procedure. Residues in gray are the template's residues corresponding to the catalytic triad

Figure S.3 Interaction maps for the four best poses of the complexes 6rw2_B (A), 3kn2_B (B) and 2obq_B (C). Protein residues are labeled in blue and peptides in green. H-bonds are highlighted in dash green lines and hydrophobic interactions are indicated by red arcs.

A

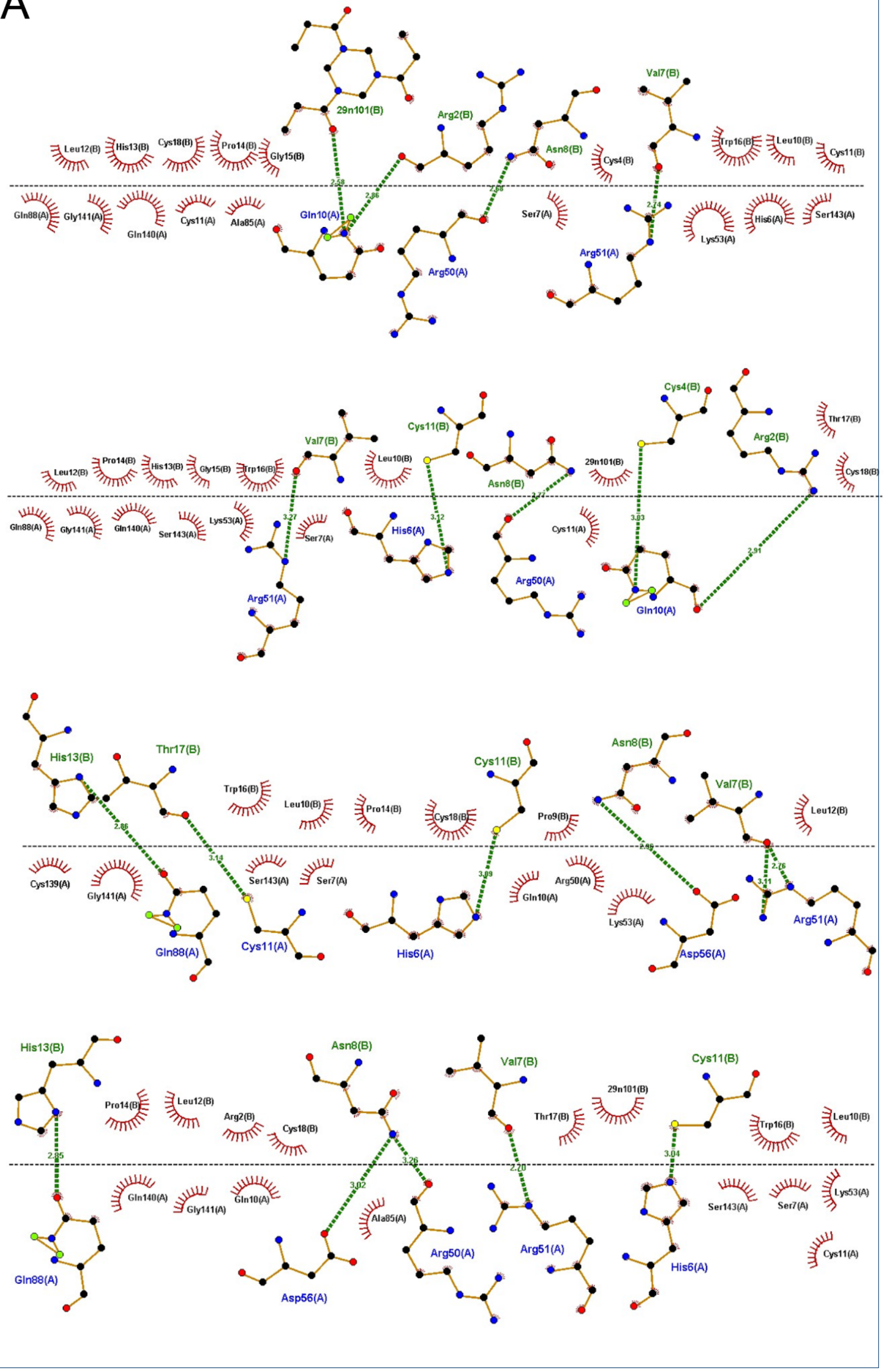

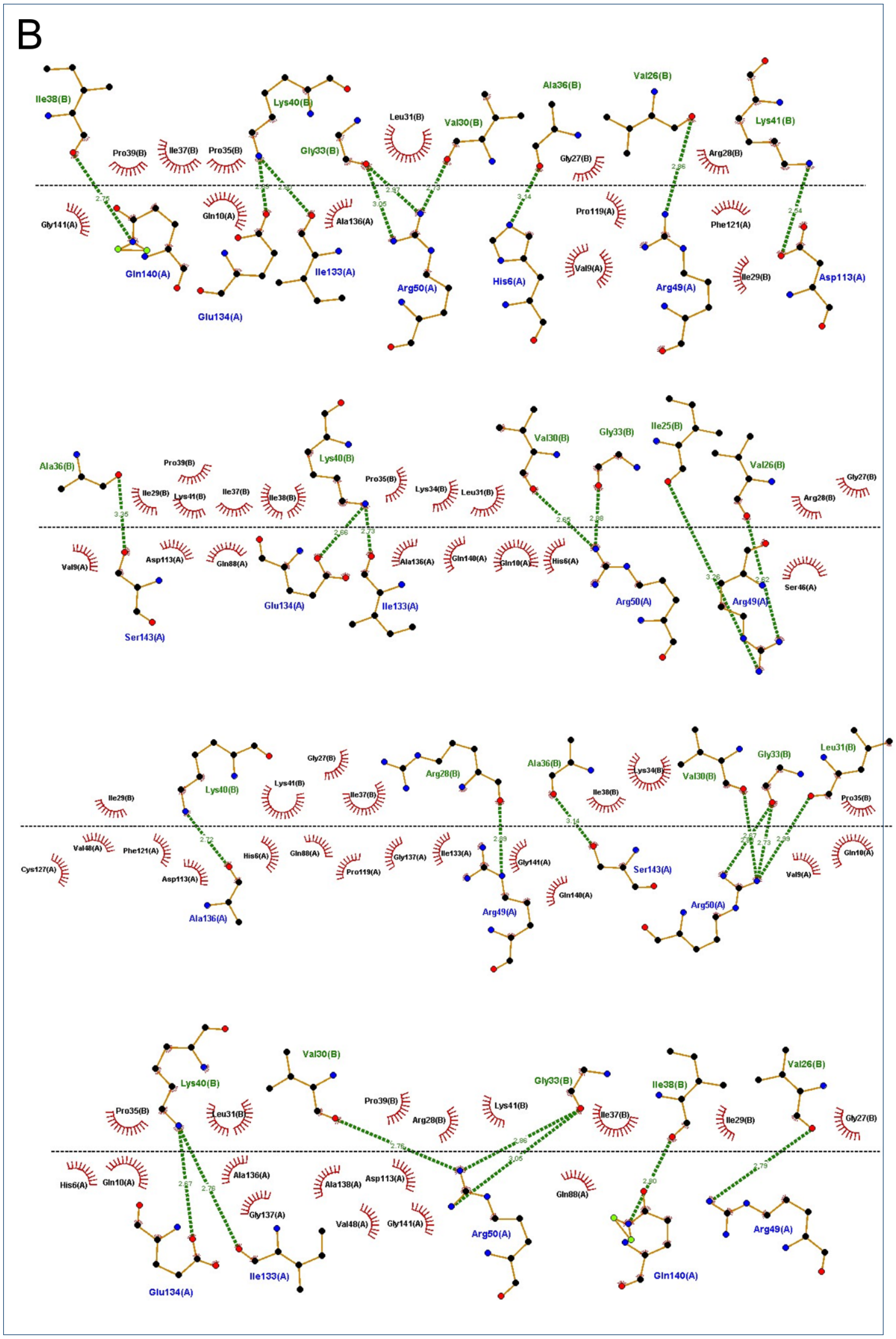
B
Ile38(B)
Lys40(B)
Leu31(B)
Val30(B)
Ala36(B)
Val26(B)
Lys41(B)
Pro39(B)
Ile37(B)
Pro35(B)
Gly33(B)
Gly27(B)
Arg28(B)
Gly141(A)
Gln10(A)
Ala136(A)
Pro119(A)
Phe121(A)
Gln140(A)
Ile133(A)
Arg50(A)
His6(A)
Val9(A)
Arg49(A)
Ile29(B)
Asp113(A)
Glu134(A)
Ile25(B)
Lys34(B)
Ile38(B)
Ser143(A)
Gln88(A)
Ser46(A)
Val48(A)
Cys127(A)
Gly137(A)
Ala138(A)

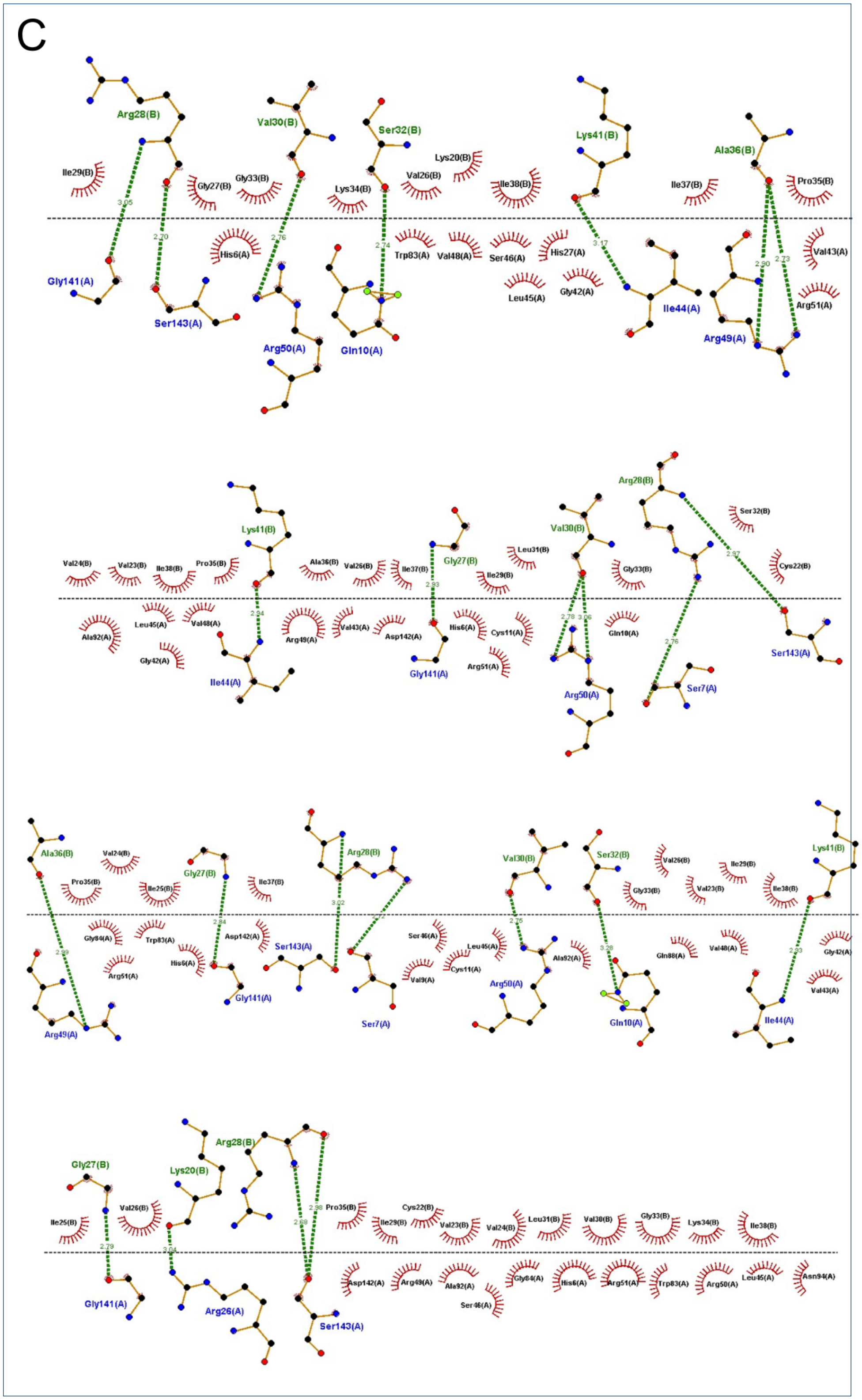
C
